# Soil Properties and Olive Cultivar Determine the Structure and Diversity of Plant-Parasitic Nematode Communities Infesting Olive Orchards Soils in Southern Spain

**DOI:** 10.1371/journal.pone.0116890

**Published:** 2015-01-27

**Authors:** Juan E. Palomares-Rius, Pablo Castillo, Miguel Montes-Borrego, Juan A. Navas-Cortés, Blanca B. Landa

**Affiliations:** Instituto de Agricultura Sostenible (IAS), Consejo Superior de Investigaciones Científicas (CSIC), Apartado 4084, 14080, Córdoba, Spain; INRA, FRANCE

## Abstract

This work has studied for the first time the structure and diversity of plant-parasitic nematodes (PPNs) infesting olive orchard soils in a wide-region in Spain that included 92 locations. It aims at determining which agronomical or environmental factors associated to the olive orchards are the main drivers of the PPNs community structure and diversity. Classical morphological and morphometric identification methods were used to determine the frequency and densities of PPNs. Thirteen families, 34 genera and 77 species of PPNs were identified. The highest diversity was found in *Helicotylenchus* genus, with six species previously reported in Spain and with *H. oleae* being a first report. *Neodolichorhynchus microphasmis* and *Diptenchus* sp., *Diphtherophora* sp., and *Discotylenchus* sp., usually considered fungal feeders, were also reported for the first time associated to olive rhizosphere. PPNs abundance ranged from 66 to 16,288 individuals/500-cm^3^ of soil with *Helicotylenchus digonicus* being the most prevalent species, followed by *Filenchus* sp., *Merlinius brevidens* and *Xiphinema pachtaicum*. Nematode abundance and diversity indexes were influenced by olive cultivar, and orchard and soil management practices; while olive variety and soil texture were the main factors driving PPN community composition. Soil physicochemical properties and climatic characteristics most strongly associated to the PPN community composition included pH, sand content and exchangeable K, and maximum and minimum average temperature of the sampled locations. Our data suggests that there is a high diversity of PPNs associated to olive in Southern Spain that can exert different damage to olive roots depending on the olive variety and their abundance. Further analysis to determine the resistance levels of most common olive varieties to the prevalent PPNs in Spain will help to choose the most appropriate ones for the establishment of new plantations. This choice will take into consideration the specific soils and environments where those olive varieties will be established.

## Introduction

Historically, and to the present times cultivated olive (*Olea europaea* L. subsp. *europaea* L.) has been culturally and economically very significant for the Mediterranean and Middle Eastern regions and remains integral to the economy of the Mediterranean area (e.g., Spain produces around 1/2 of the world production of olive oil, generating 1,886 million €) [1, 2]. Furthermore, olive orchards comprise a valuable ecological landscape determining the profitability, living and working conditions, and environmental quality of those territories. Nowadays, olive orchards cover about 10.2 M ha in the world, mainly in the Mediterranean Basin, of which more than 2.5 million ha of olives are located in Spain, mostly under rain fed production systems and 90% of them dedicated to oil production in 2012 [1, 3]. In particular, 68% of the Spanish cultivation of olives are located in Andalusia, southern Spain, occupying >1.6 M ha accounting for 19% of the total surface area of Andalusia in an impressive monoculture [1, 4].

In Andalusia, where olive production is enormously important for both economic and ecological reasons, three main olive cropping systems can be differentiated including: (i) agroforestry stands where many olive orchards are confined to slopes or rugged land, occupying large parts of mountains and hills of the Mediterranean landscape, (ii) traditional groves located in rolling plains, with gentler slopes, deeper and better soils, normally rain fed, with low plant density (less than 100 olive trees/ha), intensive tillage, low inputs in fertilizer and pesticides and manual harvest, and (iii) new intensive orchards where traditional groves are being adapted or progressively substituted by high plant density (200–400 olive trees/ha), drip-irrigated plantations, with reduced tillage, high inputs of pesticides and fertilizers and mechanical harvesting in order to push up olive yields [5, 6]. Additionally, olive production has become an example of the world-wide trend towards environmentally friendly agricultural strategies, so that new management systems such as integrated production and organic farming are being promoted to reduce negative environmental impacts of excessive use of pesticides and fertilizers. Indeed, in the last two decades it there has been a growing demand for organic olive oil which has resulted in the adoption by farmers of organic production in many traditional olive growing areas of Spain, Italy, Portugal, and Greece [7]. In Andalusia southern Spain, the cultivation of the olive under the guidelines of organic management has increased by 30% from 2006 to 2012, exceeding 54,800 ha of organic olive groves in 2012 [4], most of them (over 40%) being located in the province of Córdoba.

Some studies have shown that the shift to organic production, in parallel with an increase in use of cover crops and mechanical mowing, may enhance sustainability of olive production in the traditional olive-growing areas of Andalusia, mainly through increased soil conservation and improvement of soil physicochemical and biological properties [8–11]. However, the potential impact that those changes in the olive cropping system might have on biological soil properties, including changes in populations of soilborne pathogens of olive crop remains unknown.

Olive crop production in the Mediterranean area can be threatened by different diseases, mainly of fungal aetiology, which may result in a reduction in olive yields and plant vigor and/or longevity. Furthermore, modern olive production systems based largely on the establishment of new orchards under high-input schemes may create an environment more conducive to plant-parasitic nematode (PPN) diseases [12–14]. However, the specific negative effect on plant growth and yield by PPNs parasitism that may result from the disruption that they cause to normal processes of plant root growth and function have not been studied in detail [15]. This may be mainly due to the fact that damage to established olive orchards by nematode parasitism may be not clearly perceived by farmers since olive is an extremely vigorous plant able to thrive in relatively dry areas, which may jeopardize expression of symptoms from PPNs attacks [12]. Thus, water stress and nutrient deficiencies resulting from damage in the plant root system that can be major consequences of PPNs attacks may not be perceived by farmers or if so attributed to other soilborne plant pathogens or to poor soil quality.

Olive trees serve as hosts to a large number of PPNs, of which root-knot nematodes (*Meloidogyne* spp.), root-lesion nematodes (*Pratylenchus* spp.), spiral nematodes (*Helicotylenchus* spp.), and *Criconemoides xenoplax* are widely distributed [12, 16–18]. Conversely, limited distribution on olive has been reported for the citrus (*Tylenchulus semipenetrans*) and the cyst-forming (*Heterodera mediterranea*) nematodes [19, 20]. Some of these nematodes are recognized as pathogenic to olive (*Meloidogyne* sp., *Pratylenchus* sp., *Tylenchulus semipenetrans* and *Heterodera mediterranea*) [12, 16]. Over 150 species of PPNs have been reported in association with olive [16–18, 21], including other genera than those previously cited above, such as *Amplimerlinius* spp., *Aorolaimus* spp., *Paratrichodorus* spp., *Paratylenchus* spp., *Pratylenchoides* spp., *Trichodorus* spp., *Tylenchorhynchus* spp., *Xiphinema* spp. [17–18, 22]. However, there is a lack of information whether the PPN populations infesting olive soils apart from the phytopathological interest may be a useful bioindicator of soil health and of olive orchard or soil management systems [23]. In fact, soil disturbance has been positively correlated with the abundance and diversity of PPNs or the Plant Parasitic Index (PPI) [24]. Also, increased nutrient uptake by plants seems to cause a shift within the population composition of the PPNs [25]. Thus, knowledge of PPN species distribution, together with data on nematode population densities and structure in rhizospheric soil of cultivated olives, and the effects that farm management system may have on those populations would be useful for the management of the diseases that they might cause, as well as for helping to choose the most appropriate management system. Therefore, the objectives of this study were to determine: (i) the identity, frequency, and population density of PPNs infesting olive orchard soils in a wide area of Andalusia, Southern Spain, and (ii) to evaluate the influence that abiotic factors (including climate and soil physicochemical properties) and agronomic factors (including orchard and soil management systems, olive cultivar, irrigation regime and age of plantation) may have on the frequency and population densities and structure of those nematodes.

## Material and Methods

### Ethics Statement

No specific permits were required for the described field studies. Permission for sampling the olive orchards were granted by the landowner. The 92 olive orchards sampled in this study have been included in previous studies [10, 26] aimed to study bacterial and mycorrhizal communities and functional diversity of the olive rhizosphere. The sites are not protected in any way. The areas studied do not involve any species endangered or protected in Spain.

### Soil collection and nematode extraction

Soil samples were collected from May to July 2009 in 89 commercial olive orchards and three locations containing wild or feral forms of olive (‘Acebuches’) at southern Spain. From the commercial olive orchards, 47 are under conventional, and 42 are under organic management systems. Sampling procedures, specific location and a detailed description of the orchard sampled have recently been provided [10, 26] and also are included in S1 Table. In each olive orchard eight trees were randomly selected for soil sampling. Soil samples were collected with a shovel discarding the upper 5-cm top soil profile, from a 5- to 40-cm depth, in the close vicinity of active olive roots. This ensured that roots from weeds or other herbaceous plants were unlikely sampled. All soil samples from all trees of each olive orchard were thoroughly mixed to obtain a single representative sample per orchard before nematode extraction. This sampling strategy allowed obtaining an estimation of the most frequent PPNs that are closely associated to olive rhizosphere. Soil parameters used in the study included soil organic matter (SOM), organic C, organic N, C:N ratio, extractable P, exchangeable K, cation exchange capacity (CEC), pH (KCl), clay (%) and Sand (%) and have been reported before [26].

Nematodes from the soil were extracted from a 500-cm^3^ sub-sample using the magnesium sulphate centrifugal-flotation method [27]. Briefly, the soil was washed thoroughly with tap water through a 710-μm mesh sieve, and the filtered water was collected in a beaker and thoroughly mixed with 4% kaolin (v/v). This mixture was centrifuged at 1,100×g for 4 min, and then the supernatants were discarded. Pellets were resuspended in 250 ml MgSO4 (δ = 1.16) and the new suspensions were centrifuged at 1,100×g for 3 min. Supernatants were sieved through a 5 μm mesh, and nematodes collected on the sieve were washed with tap water, transferred to a cross-linked 8 × 8 cm square counting plate where the total number of PPNs per sample was counted under the stereomicroscope at 50xmagnification using a hand-tally counter [27]. The extracted nematodes were identified by selecting adult nematode specimens of separate genera which were fixed in 4% formaldehyde, processed to glycerin [28], and identified by morphological traits to genus or species level.

### Diversity indices

Abundance of nematodes, and the Richness, Shannon and Evenness diversity indexes were calculated using the *vegan* version 1.17–6 package [29] with the R version 3.0.1 software (R Core Development Team). Additionally, the plant parasitic index (PPI) for plant feeding nematodes was calculated according to Bongers [30] as Σ *vi* x *fi*, where *vi* is the c-p value of taxon *i* in each olive orchard listed in Table 1, and *fi* is the frequency of that taxon in a sample.

**Table 1:** Species, common-name, family, prevalence, and density (average number of individuals in 500 cm^3^ of soil) of plant-parasitic nematodes infecting 92 olive orchards in southern Spain.

**Nematode species**	**Nematode common-name**	**Family**	**Number of samples**	**Prevalence (%)**	**Average density^a^**	**Minimum ^a^**	**Maximum**	**cp^b^**	**Parasitism on olive [Reference]^b^**
Aglenchus Agricola	tylenchids	Tylenchidae	18	19.57	20.56	7	58	2	p, [12]
Amplimerlinius longicauda	stunt	Telotylenchidae	1	1.09	56.00	56	56	3	p, [12]
Amplimerlinius magnistylus	stunt	Telotylenchidae	4	4.35	25.75	3	56	3	p, [12]
Amplimerlinius paraglobigerus	stunt	Telotylenchidae	1	1.09	3.00	3	3	3	+, [12, 18]
Aorolaimus perscitus	spiral	Hoplolaimidae	3	3.26	212.33	4	621	3	+, [12, 18]
Aprutides guidetti	aphelenchids	Seinuridae	3	3.26	20.67	5	33	2	-
Basiria sp.	tylenchids	Tylenchidae	4	4.35	22.50	7	31	2	-
Bitylenchus hispaniensis	stunt	Telotylenchidae	7	7.61	415.71	7	1580	3	+, [12]
Coslenchus alacinatus	tylenchids	Tylenchidae	2	2.17	61.00	12	110	2	-
Coslenchus costatus	tylenchids	Tylenchidae	10	10.87	39.60	14	121	2	-
Criconema annuliferum	ring	Criconematidae	6	6.52	30.00	2	48	3	+, [12, 18]
Criconemella rosmarini	ring	Criconematidae	1	1.09	2.00	2	2	3	p, [12]
Criconemoides amorphus	ring	Criconematidae	7	7.61	177.00	3	910	3	+, [12, 18]
Criconemoides informis	ring	Criconematidae	11	11.96	50.09	2	324	3	+, [12, 18]
Criconemoides sphaerocephalum	ring	Criconematidae	5	5.43	157.80	4	742	3	+, [12, 18]
Criconemoides xenoplax	ring	Criconematidae	30	32.61	67.13	3	458	3	+, [12, 18, 52]
Diphtherophora sp.	dorylaimds	Diphtherophoridae	22	23.91	11.68	2	34	3	-
Diptenchus sp.	anguinids	Anguinidae	1	1.09	261.00	261	261	2	-
Discotylenchus sp.	tylenchids	Tylenchidae	1	1.09	69.00	69	69	2	-
Ditylenchus sp.	stem and bulb	Anguinidae	28	30.43	33.25	3	148	2	-
Filenchus aquilonius	tylenchids	Tylenchidae	3	3.26	76.00	21	110	2	-
Filenchus ditissimus	tylenchids	Tylenchidae	5	5.43	25.60	4	47	2	-
Filenchus sandneri	tylenchids	Tylenchidae	16	17.39	51.38	7	184	2	-
Filenchus sp.	tylenchids	Tylenchidae	59	64.13	103.32	2	1870	2	-
Filenchus thornei	tylenchids	Tylenchidae	31	33.70	94.03	10	540	2	-
Filenchus vulgaris	tylenchids	Tylenchidae	16	17.39	162.69	12	784	2	-
Helicotylenchus canadensis	spiral	Hoplolaimidae	4	4.35	4535.00	1860	10100	3	p, [12]
Helicotylenchus digonicus	spiral	Hoplolaimidae	72	78.26	1829.07	12	14200	3	+, [12, 16, 41]
Helicotylenchus dihystera	spiral	Hoplolaimidae	1	1.09	620.00	620	620	3	+, [16, 61]
Helicotylenchus exallus	spiral	Hoplolaimidae	1	1.09	91.00	91	91	3	p, [12]
Helicotylenchus oleae	spiral	Hoplolaimidae	6	6.52	1255.83	13	7100	3	+, [12, 16, 60]
Helicotylenchus pseudorobustus	spiral	Hoplolaimidae	4	4.35	671.75	40	1840	3	+, [12, 16, 41]
Helicotylenchus vulgaris	spiral	Hoplolaimidae	4	4.35	5586.00	244	14800	3	+, [12, 16, 18]
Heterodera mediterranea	Cyst	Heteroderidae	1	1.09	320.00	320	320	3	+, [12, 16, 36]
Longidorus magnus	needle	Longidoridae	2	2.17	2.00	1	3	5	p, [12]
Longidorus sp.	needle	Longidoridae	1	1.09	2.00	2	2	5	+, [12, 16, 18]
Meloidogyne arenaria	root-knot	Meloidogynidae	1	1.09	32.00	32	32	3	+, [12, 16, 41]
Meloidogyne artiellia	root-knot	Meloidogynidae	1	1.09	2980.00	2980	2980	3	-
Merlinius brevidens	stunt	Telotylenchidae	58	63.04	81.72	4	892	3	p, [12, 52]
Merlinius leptus	stunt	Telotylenchidae	1	1.09	31.00	31	31	3	p, [12]
Merlinius nanus	stunt	Telotylenchidae	1	1.09	14.00	14	14	3	p, [12]
Merlinius nothus	stunt	Telotylenchidae	1	1.09	32.00	32	32	3	p, [12]
Merlinius obscurus	stunt	Telotylenchidae	6	6.52	151.67	[12]	387	3	p, [12]
Neodolichorhynchus microphasmis	stunt	Telotylenchidae	3	3.26	410.67	[12]	610	3	p, [12]
Neopsilenchus sp.	tylenchids	Tylenchidae	1	1.09	5.00	5	5	2	-
Ogma rhombosquamatum	ring	Criconematidae	12	13.04	750.33	17	6300	3	+, [12, 16, 18]
Paratrophurus loofi	stunt	Telotylenchidae	2	2.17	1403.50	7	2800	3	-
Paratylenchus ciccaronei	pin	Paratylenchidae	2	2.17	830.50	21	1640	2	+, [12, 18, 52]
Paratylenchus microdorus	pin	Paratylenchidae	31	33.70	74.97	3	742	2	+, [12, 18, 52]
Paratylenchus sheri	pin	Paratylenchidae	9	9.78	463.89	11	2320	2	+, [12, 18]
Paratylenchus vandenbrandei	pin	Paratylenchidae	2	2.17	27.50	18	37	2	+, [12, 18]
Pratylenchus crenatus	root-lesion	Pratylenchidae	1	1.09	241.00	241	241	3	+, [12, 18]
Pratylenchus neglectus	root-lesion	Pratylenchidae	15	16.30	346.57	1	3410	3	-
Pratylenchus penetrans	root-lesion	Pratylenchidae	1	1.09	42.00	42	42	3	+, [12, 16, 41]
Pratylenchus thornei	root-lesion	Pratylenchidae	20	21.74	84.80	3	542	3	-
Psilenchus hilarulus	tylenchids	Tylenchidae	7	7.61	14.14	5	39	2	-
Psilenchus hilarus	tylenchids	Tylenchidae	1	1.09	11.00	11	11	2	-
Psilenchus sp.	tylenchids	Tylenchidae	2	2.17	9.50	9	10	2	-
Rotylenchus robustus	spiral	Hoplolaimidae	1	1.09	720.00	720	720	3	+, [16]
Trichodorus andalusicus	stubby-root	Trichodoridae	1	1.09	2.00	2	2	4	+, [12, 18]
Trichodorus giennensis	stubby-root	Trichodoridae	7	7.61	4.67	2	14	4	+, [12, 18]
Trophurus imperialis	stunt	Telotylenchidae	1	1.09	101.00	101	101	3	-
Tylenchorhynchus clarus	stunt	Telotylenchidae	14	15.22	496.07	7	3890	3	+, [12, 18]
Tylenchorhynchus dubius	stunt	Telotylenchidae	4	4.35	302.25	58	840	3	+, [12, 18]
Tylenchorhynchus maximus	stunt	Telotylenchidae	1	1.09	48.00	48	48	3	+, [12, 18]
Tylenchorhynchus mediterraneus	stunt	Telotylenchidae	7	7.61	676.14	24	1820	3	p, [12]
Tylenchorhynchus ventrosignatus	stunt	Telotylenchidae	3	3.26	55.33	32	91	3	p, [12]
Tylenchorhynchus zeae	stunt	Telotylenchidae	3	3.26	152.67	41	360	3	p, [12]
Tylenchus davainei	tylenchids	Tylenchidae	39	42.39	113.56	7	1410	2	p, [52]
Tylenchus elegans	tylenchids	Tylenchidae	13	14.13	55.92	7	134	2	-
Tylenchus hamatus	tylenchids	Tylenchidae	3	3.26	232.67	184	310	2	-
Tylenchus sp.	tylenchids	Tylenchidae	7	7.61	96.29	7	480	2	-
Xiphinema adenohystherum	dagger	Longidoridae	2	2.17	2.00	1	3	5	p, [12]
Xiphinema italiae	dagger	Longidoridae	3	3.26	15.00	1	22	5	+, [12, 16, 18]
Xiphinema nuragicum	dagger	Longidoridae	9	9.78	11.00	1	26	5	p, [12, 52]
Xiphinema pachtaicum	dagger	Longidoridae	54	58.70	32.31	3	412	5	+, [12, 16, 18]
Zygotylenchus guevarai	root-lesion	Pratylenchidae	15	16.30	106.40	10	780	3	-

^a^Average and minimum nematode levels in fields where this species was detected;^b^ Colonizer-persister value according to Bongers [30]

^b^ Nematode species are recognized as parasite (+), potentially parasite (p), or not parasite (-) of cultivated or wild olives.

### Association between the distribution of plant-parasitic nematodes from olive and abiotic and agronomic characteristic of olive orchards

Each of the 89 commercial olive orchards and 3 wild olive locations sampled were characterized for the presence, identity and frequency of PPNs. The different environmental and agronomic factors that characterize each of the 92 olive orchards and wild olive locations sampled in Andalusia were reported in a previous study [26]. The rank-based Kruskall-Wallis test was used to determine differences in all estimated diversity indexes in relation to the different agronomic factors of the olive orchards evaluated using the NPAR1WAY procedure of the Statistical Analysis System software package (SAS version 9.4; SAS Institute, Cary, NC, USA). Multiple pairwise comparisons between orchard management systems (OMS), soil management systems (SMS) and cultivar levels were determined by the Dunn test due to the unequal sample size or tied sample ranks using the KW_MC macro for SAS [31]. Correlation between diversity indexes was estimated using the Kendall tau b correlation coefficient (*Ʈ_ken,b_*) (calculated with the CORR procedure of SAS. The *Ʈ_ken,b_* was also used to estimate the correlation between abundant PPNs species.

Unsupervised cluster analysis based on the Bray-Curtis dissimilarity using the Ward’s Minimum Variance Clustering method was calculated as a preliminary step towards inferring any structure in the PPN populations among olive orchard soils sampled. The optimum number of clusters and the degree of membership of an olive orchard to its cluster was estimated on the basis of the maximum average silhouette width according to K-means partitioning. Those analyses were performed using the *cluster* package version 1.15.2 [32] with the R software.

Non-metric multidimensional scaling (NMDS) analyses were performed using MetaMDS function within the *vegan* package of R software based on dissimilarities calculated using the Bray–Curtis index obtained for data of nematode frequency, using 1,000 runs with random starting configurations, and environmental variables (agronomic and climatic characteristics and soil physicochemical properties) were fitted using the envfit routine. Also a Multivariate Regression Tree (MRT) was calculated to explore, describe, and predict relationships between multispecies data and environmental characteristics [33]. The MRT was calculated within the *mvpart* version 1.6–2 package with the R software, using the one-standard error rule on the cross-validated relative error to determine the number of terminal nodes [33].

## Results and Discussion

### Diversity and identity of Plant-parasitic nematodes infecting olive orchards

Morphological and morphometric studies of diagnostic characters allowed the identification of 13 families, 34 genera and 77 species of PPNs associated with olive orchard soils in southern Spain, including 10 species identified at genus level (*viz*. *Basiria* sp., *Diphtherophora* sp., *Diptenchus* sp., *Discotylenchus* sp., *Ditylenchus* sp., *Filenchus* sp., *Longidorus* sp., *Neopsilenchus* sp., *Psilenchus* sp., and *Tylenchus* sp.) (Table 1). Among them, spiral, stunt, pin, root-lesion and ring nematodes were the most abundant (Table 1). The highest diversity was found in the spiral nematode genus *Helicotylenchus* with 7 species, *viz*. *H. canadensis*, *H. digonicus*, *H. dihystera*, *H. exallus, H. oleae*, *H. pseudorobustus*, and *H. vulgaris* (Table 1), which were previously reported in Spain [34] with the exception of *H. oleae* which is reported associated to olive rhizosphere for the first time. Overall, the total number of PPNs in each orchard ranged from 66 (field S2) to 16,288 (field S29) individuals/500-cm^3^ soil and their percentage respect the total of PPNs in a sample ranged from 0.02% (*Trichodorus giennensis*) to 98.50% (*Helicotylenchus digonicus*) (*data not shown*). *Helicotylenchus digonicus* was the most prevalent PPN (measured as the percentage of orchards in which the PPN species was found), and was found in 78.26% of olive orchards, followed by *Filenchus* sp., *Merlinius brevidens* and *Xiphinema pachtaicum* present in 64.13%, 63.04% and 58.70% of the orchards, respectively. The frequency of the remaining species ranged from 1.09 to 42.39, with 55 of them being found in less than 10% of the sampled orchards (Table 1).

The majority of the more abundant nematode species found in this study have been associated with olive trees in previous studies [12, 16, 21]. However, some nematode genera identified in this work had not been cited previously and constitute the first report of their association to olive rhizosphere [12, 16, 21]. Those genera included the fungal feeders *Diptenchus*, *Diphtherophora*, and *Discotylenchus*, and the PPN genus *Neodolichorhynchus* [35], although these nematodes occurred rarely. Interestingly, most species considered particularly damaging PPNs were detected only in single fields and at a low density: *Heterodera mediterranea*, *Meloidogyne arenaria* and *M. artiellia*. *Heterodera mediterranea* is a highly specialized nematode associated with cultivated and wild olives forming syncytia and inducing a disorder in the stellar structures [36–37]. *Meloidogyne arenaria* has been also previously associated with olive in Spain [12, 36]. *Meloidogyne artiellia* could be associated with cultivated and wild grasses and legumes growing as cover crops in the sampled orchards rather than with olive; as olive is not a suitable host for this PPN species [38]. Four *Pratylenchus* species were identified associated to olive rhizosphere in our study (Table 1). Of them only *P. penetrans* is particularly damaging to olive. In fact, from the wide number of *Pratylenchus* species associated with olive, only this species together with *P. vulnus* have been demonstrated to be pathogenic to olive in plant bioassays in artificial inoculations [39–41]. Recently, *P. oleae* that was not observed in this work has been described infecting roots of wild and cultivated olives suffering tree decline in southern Spain and Tunisia [42].

### Diversity indexes

**Fig 1:**
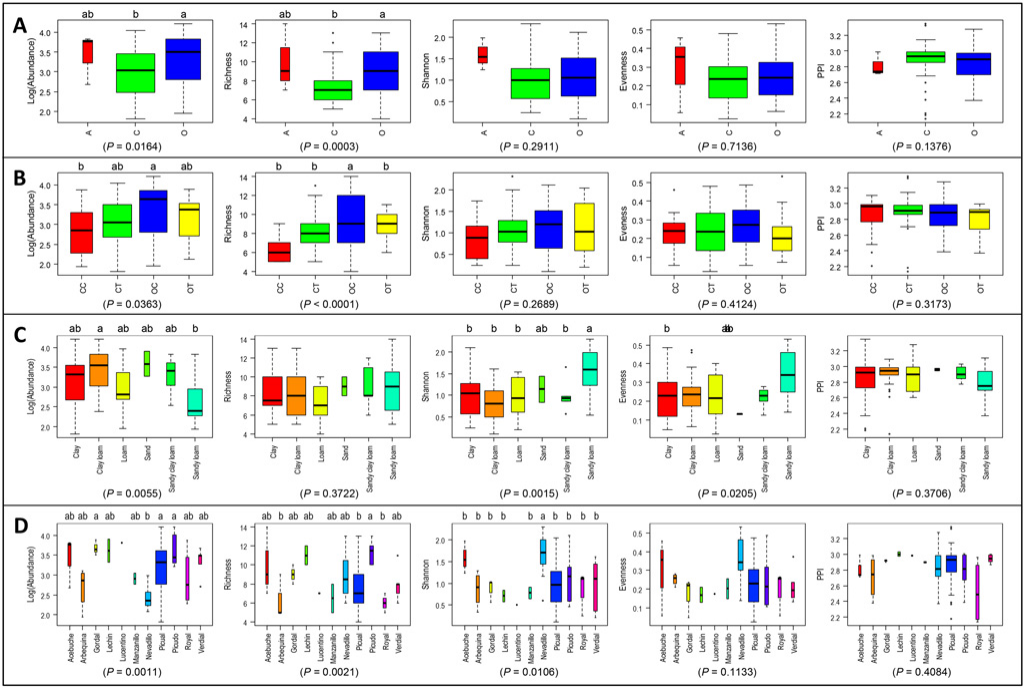
Summary box-plots of nematode abundance, Richness, Shannon, and Evenness diversity indexes and Plant Parasitic Index (PPI) derived from results of nematode identification in 92 olive orchards (S1 Table) grouped by the agronomic characteristics of the olive orchards sampled. (A) Orchard management systems included: A = Wild olives or ‘Acebuches’, O = Organic management; C = Conventional management. (B) Soil management systems included: CC = Conventional management with cover crop, CT = Conventional management with tillage, OC = Organic management with cover crop, and OT = Organic management with tillage. (C) Soil texture. (D) Olive cultivar. The rank-based Kruskall-Wallis test was used to determine differences in all estimated diversity indexes in relation to the different agronomic factors and the resulting probability values are shown. For each agronomic parameter and diversity index, boxes with a different letter indicate significant differences (*P* < 0.05) according to multiple pairwise comparisons between OMS, SMS, soil texture and olive cultivar levels determined by the Dunn test. (*) ‘Lucentino’ was present in only one orchard and was not included in the statistical analyses.

Several diversity indexes were estimated in our study (nematode abundance, Richness, Shannon and Evenness diversity indexes, and PPI; Fig. 1, S1 Table) and tested for differences associated to the agronomical characteristics of the olive orchards sampled (Fig. 1). Overall, abundance of PPNs was significantly and directly correlated (*Ʈ_ken,b_* >0.2255, n = 92, *P* < 0.0016) with the Richness and PPI indexes, while this correlation was inverse (*Ʈ_ken,b_* < -0.1822, n = 92, *P* < 0.0100) with the Shannon and Evenness indexes (*data not shown*). Soil species diversity tends to increase with increasing resource availability, which may explain that those samples supporting higher abundance of PPNs show higher Richness and PPI indexes [43]. Globally, the PPN abundance and Richness indexes were more effective in detecting significant differences (*P* < 0.05) between levels of the studied agronomic characteristics.

The OMS significantly (*P* < 0.05) influenced PPN abundance and Richness index, while no significant differences (*P*≥0.05) occurred for the remaining three diversity indices tested (Fig. 1A,B). The PPN abundance was significantly higher (*P* = 0.0164) in orchards managed organically (O) compared to that managed conventionally (C), and the opposite occurred for the Richness index (*P* = 0.0003). Wild olives (A), showed intermediate values for both PPN diversity indexes that did not differ (*P*≥0.05) from those under O or C management (Fig. 1A). Our results obtained for PPN species are in agreement with that obtained by García-Ruiz et al. [44] for a limited set of olive orchards in which they analyzed the global nematofauna (including both free-living and PPN nematodes). Only one orchard from three sites sampled in Andalusia showed differences due to the management (organic *versus* conventional) for mean Richness index of plant and unicellular eukaryote feeding and omnivorous nematodes [44]. In previous studies focused in the same olive orchards sampled here we found that in general olive orchards under organic management showed higher microbial diversity in the rhizosphere as compared to conventionally managed orchards [10], however the structure and diversity of arbuscular mycorrhiza in the olive rhizosphere did not differ among OMS [26]. These findings could be the result of the influence of the herbaceous plants that compose the soil cover which may affect PPNs at the species level as suggested by Neher [45], since plant functional groups (i.e., grasses, legumes) have contrasting rooting patterns that create habitats more favorable to some species of PPNs such as ecto-parasites. Furthermore, SOM, organic C, organic N, C:N ratio were significantly higher in organic as compared to conventional management in the same orchards of this study [10]. Our results agree with previous reports of the existence of a higher number and diversity of PPNs in fields managed organically [46–49], however the lack of clear differences in abundance or diversity indexes of soil biota in other studies is attributed to the fact that soil type in general had a much stronger effect on the soil biota compared to orchard or farm management type [48, 50–51].

Soil management has a similar trend than OMS (Fig. 1B). Among SMS, the highest abundance (*P* = 0.0363) and Richness of PPN (*P* < 0.0001) occurred in organic olive orchards with the presence of a cover crop (OC), decreasing (*P* < 0.05) in orchards under either organic (OT) or conventional management with tillage (CT); being significantly lowest (*P* < 0.05) in orchards under conventional management with the presence of a cover crop (OC) (Fig. 1B). In our study, the presence of a cover crop in organic managed orchards might have increased the number and diversity of PPNs probably due to the presence of a food source during the whole crop season and the existence of different niches in the soil in a perennial crop as olive, whereas in the conventional orchards the application of herbicide to control weeds in late spring to early summer might have had a detrimental effect on PPN populations [52]. Nevertheless, it should be noticed that PPN species can respond differently to tillage. Thus, some PPNs genera have shown a great sensitivity to soil tillage, at least immediately after its application (i.e. *Pratylenchus*, *Meloidogyne*) [53]. This might explain that several studies have found different effects of tillage on nematode abundance and diversity [53–56]. Furthermore, it should be taken into account that tillage or the presence of a cover crop not only affect PPNs directly, but also have indirect effects by impacting on natural enemies that could regulate nematode populations [53]. Since the use of cover crops have been recently introduced to minimize soil erosion, a major problem of olive orchard soils [7–8], care should be taken to avoid cover crops that might enhance or contribute to maintain PPN populations detrimental to olive. On the other hand selection of specific cover crops have been shown as a promising technique in organic farming for controlling populations of PPNs and improving soil properties [57].

Soil texture modified both, abundance (*P* = 0.0055) and diversity (Shannon and Evenness indices) (*P* < 0.0206) of PPNs but with opposite trends (Fig. 1C). Indeed, although nematode abundance was significantly higher in clay loam soils, their higher diversity was reached on sandy loam soils that showed the lower nematode abundance (Fig. 1C). No significant differences (*P* > 0.3707) were observed for the Richness and PPI indexes for the different soil textures evaluated. PPNs population densities and diversity have been shown to be significantly affected by soil texture, with lower abundances in loam than in silt soils [49] or higher diversity in soils with higher sand content [58].

Olive cultivar has been shown as an important factor determining nematode assemblages in southern Spain [52]. In this study we confirmed this finding with a sampling of a high number of olive cultivars that were grown in commercial orchards in different geographical areas of Andalusia. Olive cultivar also had a strong influence on both, PNN abundance (*P* = 0.0011) and diversity estimated by the Richness and Shannon indexes (*P* < 0.0107) although ranking of olive varieties changed across indexes (Fig. 1D). Thus, PNN abundance was significantly highest (*P* < 0.05) for ‘Picudo’, ‘Picual’ and ‘Gordal’, with ‘Nevadillo’ showing the lowest (*P* < 0.05) (Fig. 1D). The Richness index was significantly highest (*P* < 0.05) in ‘Picudo’, with ‘Picual’, ‘Royal’ and ‘Arbequina’ reaching the lowest values (*P* < 0.05). On the other hand, the Shannon index was highest in ‘Nevadillo’ followed by ‘Acebuche’, being lower in all other olive varieties that showed similar (*P*≥0.05) values among them (Fig. 1D). No significant differences (*P* > 0.1132) occurred for the Evenness and PPI indexes due to olive cultivar (Fig. 1D). Very different responses of resistance/tolerance and susceptibility to PPNs have been described in olive cultivars for specific PPNs species [12, 16]. However, although plant root vigor and exploration ability could be an important factor determining the resistance levels for PPNs in olive varieties or cultivars, the soil physicochemical characteristics and climatic conditions where the olive orchards are established can exert a higher influence on PPN populations than the genotype itself, which should be explored.

Finally, irrigation regime and crop age did not influence statistically (*P* > 0.05) any of the diversity indexes tested, with the only exception of the Shannon index that was significantly higher (*P* = 0.0116) in irrigated olive orchards compared to that under rain-fed regime (*data not shown*). Our findings support previous results indicating that soil moisture may influence diversity of PPNs in a positive way [59].

### Unsupervised analysis of the structure of plant-parasitic nematode populations

**Fig 2:**
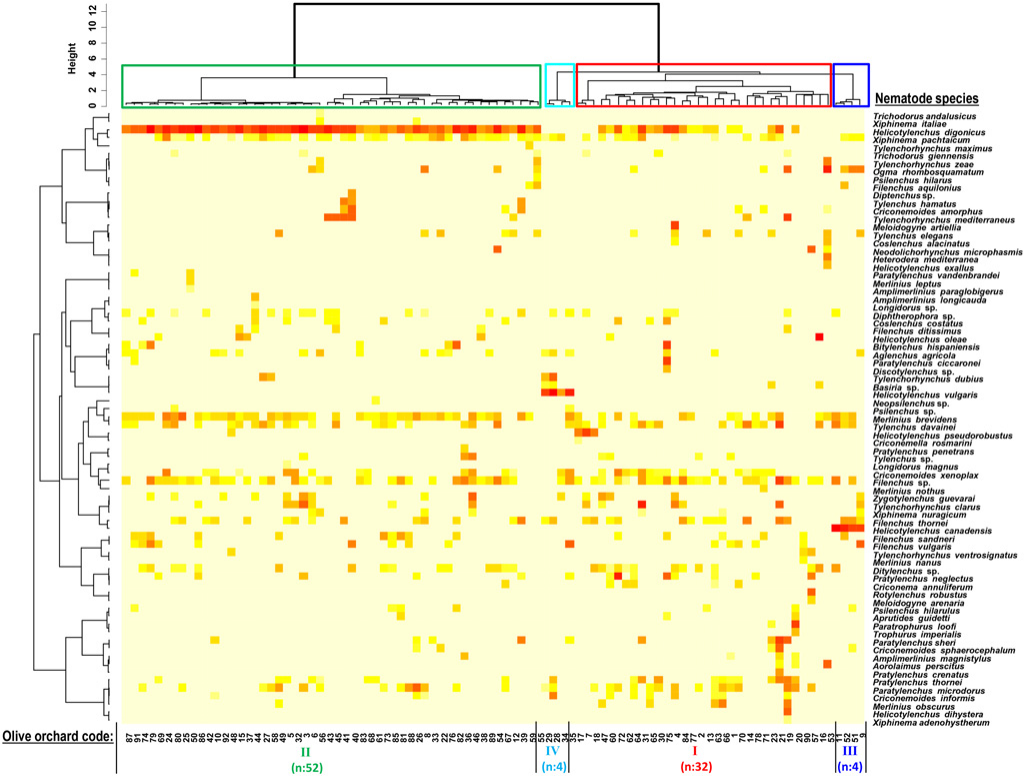
Unsupervised cluster analysis of PPN populations in olive orchards in southern Spain. The Ward linkage method was applied to the Bray–Curtis dissimilarity matrix calculated from frequency of occurrence of the 77 species of PPNs identified among the 92 olive orchard soils sampled. The optimum number of clusters and the degree of membership of an olive orchard to each of the four clusters was estimated on the basis of the maximum average silhouette width according to K-means partitioning (S1 Fig.). The intensity of color (from light yellow to deep red) shown for each nematode species correlates with abundance Log(number of individuals/500 cm3 of soil).

Unsupervised cluster analysis was performed using the Bray-Curtis dissimilarity index of PPN populations as an initial step towards inferring any structure in the PPN populations among the 92 olive orchard soils sampled. The optimum number of clusters was estimated to be four on the basis of the maximum average silhouette width obtained with K-means partitioning (Fig. 2; S1 Fig.). Olive orchards were well-clustered in their respective groups (i.e., large silhouette values), with the exception of 20 of 32 olive orchards included in cluster I that were not clearly assigned to this cluster (i.e., negative silhouette values) (S1 Fig.). *Helicotylenchus* spp. were the PPN genera with the highest influence in determining the olive orchard clustering (Fig. 2). Up to seven *Helicotylenchus* species were found being highly prevalent and abundant in the majority of the Andalusian olive orchards sampled in our study (Table 1). In all soil samples (52) from orchards in Cluster II *H. digonicus* showed the highest density, occasionally four samples included *H. oleae* and one sample included *H. pseudorobustus*, but specimens of the other four *Helicotylenchus* species were not found. Other nematode species in this cluster were less frequent and included *Merlinius brevidens* (41 orchards), *Xiphinema pachtaicum* (36 orchards), *Filenchus* sp. (32 orchards), and *Tylenchus davainei* (21 orchards). Orchards in Cluster III showed high densities of *H. canadensis*, *Ogma rhombosquamatum*, *Filenchus thornei*, *Xiphinema pachtaicum*, *Merlinius brevidens* and *Tylenchus davainei*. In contrast, soil samples from orchards included in Cluster IV showed high population levels of *H. vulgaris*, and other abundant species included *Xiphinema pachtaicum* and *Aglenchus agricola*. Cluster I included olive orchards showing a high diversity of nematode species and up to five species of *Helicotylenchus*. Other species present in high frequency in this Cluster were *Filenchus* sp. (23 orchards), *Paratylenchus microdorus* (15 orchards), *Criconemoides xenoplax* (15 orchards) and *Tylenchus davainei* (14 orchards). The influence and prevalence of *Helicotylenchus* species in olive orchards is notorious and species seem to be mutually excluded in the same field. Indeed, the frequency of occurrence of *H. digonicus* was significantly and negatively correlated (*Ʈ_ken,b_* < -0.2408, n = 92, *P* < 0.0055) with that of *H. vulgaris* and *H. canadensis*, but not with that of *H. pseudorobustus* (*Ʈ_ken,b_* = -0.1137, n = 92, *P* < 0.1908).

Some *Helicotylenchus* species have been associated with root necrosis and have been shown capable to affect olive trees growth under certain growing conditions [60]. In general, *Helicotylenchus* spp. can adopt a semiendoparasitic feeding behavior on olive feeder roots [12] and with this strategy occupy the most viable site in the root. Although olive seems to be well adapted to these parasites, a 78% plant-growth reduction has been found on olive plants inoculated with 1,000 individuals of *H. dihystera* under controlled conditions experiments [61]. Sampling sites that included feral forms of olives or ‘Acebuches’ (sites 19, 31 and 32) were distributed in different clusters [cluster II (site 32) and cluster I (sites 19 and 31)]. The diversity of site 19 (with 14 species including *H. dihystera*) was higher than that of sites 31 and 32 with 7 and 9 species, respectively, but all of them included *H. digonicus*.

From the 20th most prevalent nematode species, i.e., those present in at least 10 olive orchards, some significant (*P* < 0.05) correlations were detected: (i) *P. microdorus* was positively (*P* < 0.05) correlated with *P. thornei*, *C. informis*, *C. annuliferum*, *M. obscurus*, and *Z. guevarai*; (ii) *C. xenoplax* was positively (*P* < 0.05) correlated with *Filenchus* sp., *P. thornei* and *C. annuliferum*; (iii) *X. pachtaicum* was positively (*P* < 0.05) correlated with *O. rhombosquamatum*, but negatively (*P* < 0.05) with *M. brevidens* and *Ditylenchus* sp. In addition, some other pairwise significant correlation occurred. In samples included in cluster II a significant and negative association (*Ʈ_ken,b_* < -0.2198, n = 52, *P* = 0.0259) of *H. digonicus* was found with *X. pachtaicum*. *Xiphinema pachtaicum* is a dagger nematode widely distributed in the Iberian Peninsula in undisturbed soil with not high ecological requirements [62–63]. *H. digonicus* and *X. pachtaicum* have a semiendoparasitic feeding behavior in olive feeder roots and they might compete for occupying most viable sites in the olive rhizosphere [12].

### Factors shaping the distribution and diversity of plant-parasitic nematodes in olive orchards

**Fig 3:**
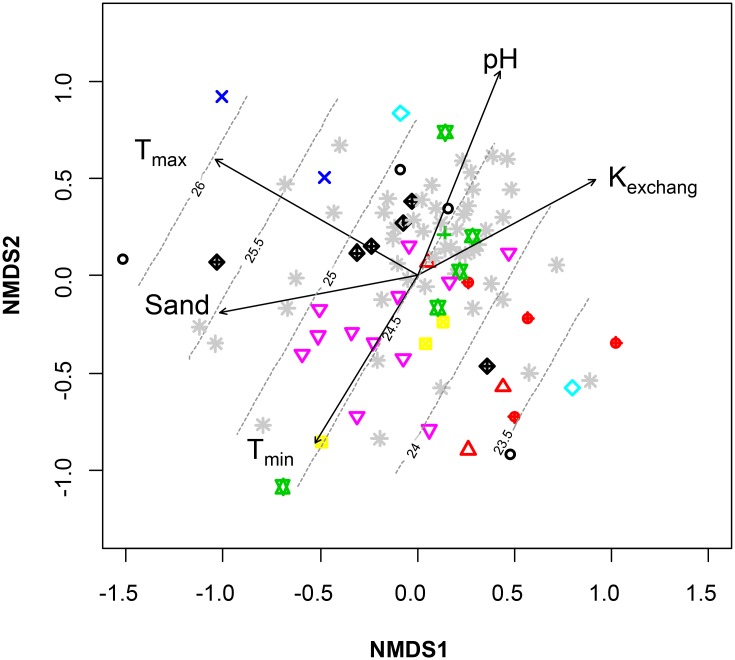
NMDS biplot of a Bray-Curtis dissimilarity matrix of nematode community analysis. The fitted vectors of environmental variables (soil physicochemical and climatic) and the agronomic variable olive cultivar (each of the 11 olive cultivars is shown with different symbols) that were most significantly and strongly associated (*P* < 0.05) with the ordination and shown in Table 2 are also represented (a generalized additive model fitted contours show also maximum temperature (Tmax) ramp (ºC).

It has been shown that although diversity indexes (such as Richness, Eveness and Shannon used in our study) and unsupervised cluster analysis are useful in describing community characteristics they do not provide information about relevant compositional features of PPNs communities taxa and the environmental factors shaping their population structure. Consequently, to specifically determine the PPN community composition and to identify hypothetical gradients likely related to the differentiation in PPN composition among the olive orchards sampled we used NMDS ordination to represent, in two dimensions, the pairwise Bray-Curtis dissimilarities between PPN frequencies (incorporating taxon abundance and identity). The projection of each of the environmental and agronomic variables independently onto the NMDS ordination (Fig. 3; Table 2) allowed to identify that within the agronomic variables PPN communities can be significantly (*P* < 0.05) differentiated according to soil texture and the cultivar genotype of the olive orchard, whereas not significant (*P*≥0.05) grouping could be found according to the orchard management system, presence of a vegetative cover, age of plantation and the irrigation regime (Table 2). Interestingly, with the exception of OMS, these later agronomic variables also did not modify abundance and diversity indexes associated to the PPN populations in the olive orchards. Thus, there was a tendency to locate olive samples in the NMDS ordination according to the olive cultivar (Fig. 3). The effect of olive genotype in soil nematode community has already been addressed above. A recent study [52] demonstrated that olive genotypes significantly influence the nematode assemblages present in their rhizospheric soil in a collection of olive cultivars growing in a single orchard and therefore under the same environmental conditions. In artificial inoculations ‘Picual’ and ‘Arbequina’ have been tested against *C. xenoplax*, *H. digonicus*, *H. pseudorobustus*, *M. arenaria* race 2, *Meloidogyne incognita* race 1, *Meloidogyne javanica*, *Pratylenchus penetrans* and *Pratylenchus vulnus* and both cultivars showed differences in reproduction factors for the two *Helicotylenchus* spp. [41]. Other studies have found differences between cultivars and olive rootstocks in the reproduction of *Meloidogyne* spp., *P. vulnus* and *Xiphinema index*, in some cases associated with inoculum levels [21, 64]. We hypothesize that several root parameters (size, numbers, softness, exudation) could affect the diversity and abundance of PPN species in the rhizospheric soil. Also, specific bacterial, fungal and mycorrhizal rhizosphere populations associated to specific olive cultivars could affect the plant attractiveness and nematode pathogenicity [8, 65–67]. This effect could be even stronger in crops with a long period of establishment in the field as it is the case for olive.

**Table 2:** Summary of relationships between agronomic, soil and environmental factors and plant-parasitic nematode communities in a collection of 92 olive orchards in Southern Spain^a^.

**Factors ^b^**	***r^2^***	***P***	
Soil physicochemical variables			
Clay (%)	0.0238	0.36863	
**Sand (%)**	0.0806	0.03297	*
Organic C (%)	0.0094	0.64835	
Organic N (%)	0.002	0.93107	
Extractable P (ppm)	0.0169	0.50350	
**Exchangeable K (ppm)**	0.0817	0.02897	*
CEC	0.0005	0.97602	
C:N ratio	0.0656	0.05095	.
**pH(KCl)**	0.0965	0.01399	*
SOM (%)	0.0094	0.65035	
**Soil texture**	0.2114	0.00199	**
Climatic variables			
Total Rainfall	0.0344	0.20679	
Average Rainfall	0.0234	0.33866	
ETP	0.0204	0.38761	
**Tmax**	0.1077	0.00699	**
**Tmin**	0.0771	0.03297	*
Tmean	0.0386	0.18581	
Altitude	0.0330	0.20380	
Agronomic variables			
**Olive cultivar**	0.2334	0.00199	**
Presence of vegetative cover	0.0068	0.54046	
Age of plantation	0.0173	0.54845	
Irrigation regimen	0.0271	0.08492	
Orchard management system	0.0354	0.16883	

^a^Correlations with soil physicochemical, environmental and agronomic variables (*r* ^2^) were obtained by fitting linear trends to the NMDS ordination obtained in Fig. 3 and significance (*P*) was determined by permutation (nperm = 1000). ‘***’ = *P* < 0.001; ‘**’ = *P* < 0.01; ‘*’ = *P* < 0.05; ‘.’ = *P* < 0.1. Variables with highest significant weight are shown in bold.

^b^Orchard agronomic characteristics, and climatic and soil physicochemical properties were reported before [10, 26].

The host plant has been shown as the most important factor driving force in nematode populations, but abiotic factors are also important in maintaining the steady state [58]. PPNs composition among olive orchards was also strongly related (*P*<0.0330; 0.233>*r^2^*>0.077) to several environmental and agronomic characteristics comprising maximum and minimum temperature, soil texture, soil pH, and exchangeable K and sand content (Fig. 3, Table 2). Other environmental factors showing a lower effect (*P* < 0.051) included the C:N ratio of the soil samples (Table 2).

**Fig 4:**
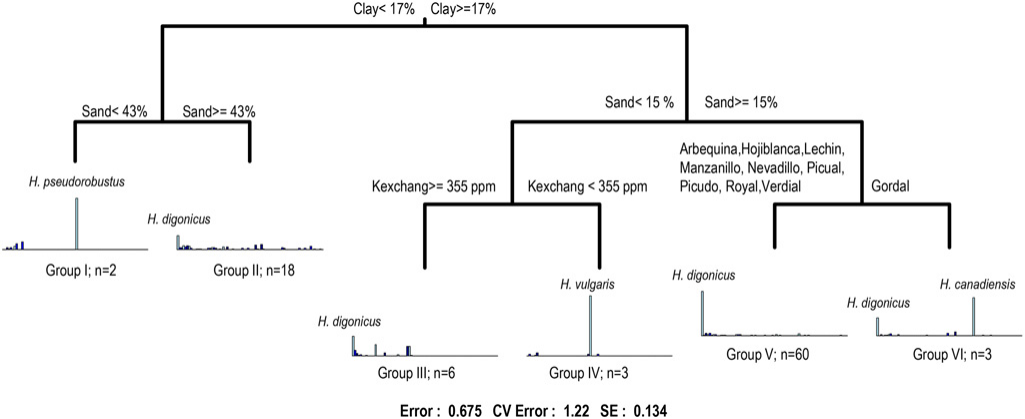
Sums of squares multivariate regression tree summarizing olive PPN community–agronomic, climatic and soil factors relationships. The tree was calculated using frequency of PPNs in each olive orchard. For each split a rule is selected based on the predictors to minimize the dissimilarity within the plant parasitic nematode profiles in the resulting two nodes (main rule is shown above the node). At each terminal node, the mean relative abundances of each plant parasitic nematode are shown as bar plots, together with the number of olive orchards for each group and the PPN species with the highest frequency.

A multivariate regression tree was also calculated to summarize the relationships between PPN community composition and environmental and agronomic variables. This tree with the most informative variable in each split is shown in Fig. 4. The tree explained >32.5% of the variability in PPN profiles, much of which were accounted by the first split based on clay content (Fig. 4). Then, sand content was the next best predictor for the second-order splits, that allowed to differentiate two groups within soils with < 17% of clay content, one (Group I) formed by two soils with high frequencies of *H. pseudorobustus* and total Richness (8 PPN species) and Group II formed by 18 soils with a higher total Richness of PPNs (46 species) including the most abundant species *H. digonicus*. Exchangeable K allowed differentiating heavier soils (clay > 17% and sand <15%), with two groups formed by Group III with six soils and moderate Richness of PPNs (23 species) and Group IV formed by three olive orchards with lower Richness (14 species) and a high frequency of *H. vulgaris*. On the other hand, for the other second-order split the three unique orchards with the olive cv. ‘Gordal’ were clearly differentiated (Group VI) from the remaining orchards (Group V, 60 soils) including soils with nine olive cultivars, a high frequency of *H. digonicus* and high total Richness (63 species). These three olive orchards from Group VI showed a lower total Richness (17 species) but a high frequency of *H. digonicus* and *H. canadensis* (Fig. 4).

Some studies have been performed in order to link crop management, soil characteristics and nematode communities [44, 48, 68–69]. Our study was carried out specifically on PPNs in a wide-region sampling area of olive orchards in Andalusia. Habitat structure is complex in soil because of a combination of physical constraints imposed by pore structure, varying soil moisture, and resource distribution (plant roots and organic debris) [45] that can interact differentially according to soils, climate, crops and geographic locations. However, available information comes from studies focused on a limited number of locations. In addition, our study is pioneer in discerning how PPNs interact specifically with the olive crop under a wide range of agronomic, climate and soil physico-chemical characteristics in a wide region in Southern Spain representative of the highest surface dedicated to olive cultivation in the world [2].

Soil type and texture have been demonstrated to have much stronger effect on PPN populations than orchard management when comparing conventional and organic farms [48] and also have strong influence in the rhizosphere microbiota of olive soils [8, 10, 26, 44]. In the same way, clay and sand content as well as average maximum and minimal annual temperatures were significantly (*P* < 0.05) associated with the PPN communities in this study, and could differentiate population structure of PPNs in olive orchards by their frequency of occurrence in the NMDS and MRT analyses. Abundance of PPNs has been positively related to mean annual temperatures in a global scale [42]. Sand content can influence the PPN communities, probably due to its indirect effect on soil pH and soil temperature. Sandy soils have higher thermal conductivity than soils with higher clay contents which may explain also their significant relationship with PPN assemblages [70]. Soil nematode communities have been shown to be highly influenced by soil pH in other studies [47, 69, 71] as well as exchangeable K that has been found as an important parameter for determining populations of some PPN genera [72]. Additionally, many different soil physicochemical characteristics (including the ones included in this study) have been associated specifically to some genera and to density gradients of some PPNs [72–73]. However, the effect of each soil factor varied according to the PPN species. How the physicochemical properties of the soil interact with PPNs are difficult to explain in some circumstances, as exchangeable K, pH and sand, have a direct effect on plant health, and this effect could be stronger that the direct effect on nematode populations [72], which deserves more research.

### Conclusions

A healthy soil is defined as a stable system with resilience to stress, high biological diversity, and high levels of internal nutrient cycling [74]. PPNs are a major constraint for agriculture that in most cases are considered as the “unseen enemies of crops” because of the unspecific visible symptoms they cause on crops (chlorosis, less vigor, early senescence, etc.) and the difficulties of their diagnosis [75]. This work has studied for the first time the population density, structure and diversity of PPNs infecting olive in a wide-region in southern Spain that included 92 locations. Our study allowed determining which agronomic or environmental factors associated to the olive orchards are the main drivers of the PPN population density and structure. Some soil physicochemical factors (texture, pH, sand and clay, and extractable K), climatic variables (minimum and maximum temperatures) and the agronomic variable olive cultivar were the factors driving the PPN population levels and community structure. Although it was restricted to a specific nematode trophic group (i.e., the plant-parasitic nematodes), this study could be of help to choose the most appropriate olive cultivar for the establishment of new plantations. This will need to take into consideration the susceptibility level of those cultivars to the PPNs present in the specific soils and environments where the plantations will be established.

## Supporting Information

S1 Table Geographic coordinates, orchard management system, soil management, olive cultivar, abundance, Richness, Shannon, and Evenness diversity indexes and Plant-Parasitic Index (PPI) of the 92 olive orchards sampled. (PDF)

S1 Fig Silhouette plot calculated using K-means partitioning showing the silhouette width for each individual sample. The number of clusters was estimated as that giving the largest average silhouette width for the 92 orchards plots.
(TIF)
