# Supplementary material for: Soil Properties and Olive Cultivar Determine the Structure and Diversity of Plant-Parasitic Nematode Communities Infesting Olive Orchards Soils in Southern Spain
Source: PLoS One. 2015 Jan 27;10(1):e0116890. doi: 10.1371/journal.pone.0116890 (PMC4308072; doi:10.1371/journal.pone.0116890)
Supplement: S1 Table — (PDF) [file pone.0116890.s001.pdf]

Orchard	Geographic location: Latitude	Geographic location: Longitude	OMS[a]	SMS[b]	Variety	Abundance	Richness	Shannon	Evenness	PPI
S1	37 ° 46 ´ 12.107 ´´N	4 ° 39 ´ 39.711 ´´W	O	OT	Picual	133	7	1.70	0.39	2.37
S2	37 ° 40 ´ 51.568 ´´N	4 ° 25 ´ 31.668 ´´W	C	CT	Picual	66	5	1.50	0.35	2.18
S3	37 ° 41 ´ 10.736 ´´N	3 ° 55 ´ 16.250 ´´W	O	OC	Picudo	15937	12	0.46	0.11	2.99
S4	37 ° 50 ´ 11.732 ´´N	3 ° 3 ´ 48.816 ´´W	O	OC	Picual	6639	13	1.19	0.27	2.97
S5	37 ° 50 ´ 5.148 ´´N	3 ° 6 ´ 3.854 ´´W	O	OC	Picual	3273	12	0.63	0.15	2.99
S6	37 ° 47 ´ 57.833 ´´N	3 ° 2 ´ 13.017 ´´W	C	CT	Picudo	2051	11	0.58	0.13	3.00
S7	37 ° 49 ´ 12.034 ´´N	3 ° 6 ´ 2.677 ´´W	C	CC	Picual	1988	8	0.38	0.09	2.97
S8	37 ° 50 ´ 53.245 ´´N	3 ° 5 ´ 51.031 ´´W	O	OC	Picudo	1646	12	1.38	0.32	2.88
S9	37 ° 52 ´ 24.991 ´´N	4 ° 52 ´ 31.767 ´´W	C	CT	Picudo	3328	10	1.27	0.29	2.74
S10	37 ° 52 ´ 24.710 ´´N	4 ° 52 ´ 33.111 ´´W	O	OT	Picual	2173	9	0.47	0.11	2.93
S11	37 ° 53 ´ 28.541 ´´N	4 ° 51 ´ 49.169 ´´W	C	CT	Picual	11071	6	0.42	0.10	2.93
S12	37 ° 46 ´ 6.732 ´´N	4 ° 39 ´ 38.977 ´´W	C	CC	Royal	300	7	1.15	0.27	2.77
S13	37 ° 42 ´ 33.743 ´´N	4 ° 18 ´ 8.000 ´´W	C	CC	Royal	189	5	1.12	0.26	2.21
S14	37 ° 42 ´ 35.065 ´´N	4 ° 18 ´ 5.859 ´´W	C	CT	Royal	1054	6	1.10	0.25	2.14
S15	37 ° 42 ´ 34.754 ´´N	4 ° 18 ´ 4.628 ´´W	C	CC	Picual	3743	7	0.40	0.09	2.98
S16	37 ° 42 ´ 32.434 ´´N	4 ° 18 ´ 3.117 ´´W	C	CC	Royal	7450	6	0.25	0.06	2.96
S17	37 ° 42 ´ 13.476 ´´N	4 ° 18 ´ 9.932 ´´W	O	OC	Manzanillo	590	5	0.65	0.15	2.89
S18	37 ° 39 ´ 37.623 ´´N	4 ° 12 ´ 55.609 ´´W	C	CC	Arbequina	530	5	1.06	0.25	2.60
S19	37 ° 39 ´ 55.872 ´´N	4 ° 12 ´ 3.542 ´´W	A	OC	Acebuche	5896	14	1.98	0.46	2.71
S20	37 ° 38 ´ 39.051 ´´N	4 ° 13 ´ 24.604 ´´W	O	OT	Picual	3384	11	0.76	0.17	2.92
S21	37 ° 39 ´ 2.932 ´´N	4 ° 13 ´ 30.750 ´´W	O	OC	Picudo	10064	13	2.11	0.49	2.39
S23	37 ° 38 ´ 10.801 ´´N	4 ° 29 ´ 7.515 ´´W	C	CT	Picual	1108	7	1.24	0.29	3.35
S24	37 ° 41 ´ 31.298 ´´N	4 ° 12 ´ 12.675 ´´W	O	OT	Picual	388	10	2.04	0.29	2.71
S25	37 ° 55 ´ 59.724 ´´N	4 ° 48 ´ 22.986 ´´W	O	OC	Picual	7965	6	0.75	0.47	3.09
S26	37 ° 54 ´ 45.659 ´´N	4 ° 48 ´ 54.588 ´´W	C	CC	Lucentino	6492	7	0.50	0.17	2.99
S27	37 ° 51 ´ 13.986 ´´N	3 ° 1 ´ 19.468 ´´W	O	OT	Picudo	2350	10	1.05	0.11	2.68
S28	37 ° 20 ´ 24.575 ´´N	3 ° 53 ´ 20.997 ´´W	O	OT	Picual	6770	6	0.38	0.24	2.98
S29	37 ° 55 ´ 33.414 ´´N	3 ° 6 ´ 32.115 ´´W	O	OC	Picual	16288	10	0.43	0.09	2.99
S30	38 ° 26 ´ 23.822 ´´N	2 ° 46 ´ 2.227 ´´W	C	CT	Picual	2311	7	0.25	0.10	2.99
S31	38 ° 26 ´ 21.258 ´´N	2 ° 46 ´ 1.864 ´´W	A	OC	Acebuche	483	7	1.54	0.06	2.73

	Geographic location									
Orchard	Latitude	Longitude	OMS[a]	SMS[b]	Variety	Abundance	Richness	Shannon	Evenness	PPI
S32	38 ° 26 ´ 23.918 ´´N	2 ° 46 ´ 1.236 ´´W	A	OC	Acebuche	6576	9	1.24	0.36	2.99
S33	38 ° 26 ´ 31.952 ´´N	2 ° 46 ´ 11.894 ´´W	C	CT	Picual	3772	7	0.56	0.29	2.96
S34	37 ° 38 ´ 10.139 ´´N	4 ° 29 ´ 5.952 ´´W	C	CT	Picual	582	5	0.97	0.13	3.32
S35	38 ° 26 ´ 30.561 ´´N	2 ° 46 ´ 14.167 ´´W	C	CT	Picual	368	6	1.08	0.22	3.14
S36	38 ° 23 ´ 49.713 ´´N	2 ° 44 ´ 57.091 ´´W	O	OC	Picual	6603	11	0.87	0.25	2.84
S37	38 ° 23 ´ 50.427 ´´N	2 ° 44 ´ 57.377 ´´W	O	OT	Picual	3126	8	0.97	0.20	2.89
S38	38 ° 25 ´ 35.736 ´´N	2 ° 44 ´ 12.183 ´´W	C	CC	Picual	1383	6	0.55	0.22	2.97
S39	38 ° 25 ´ 37.262 ´´N	2 ° 44 ´ 12.755 ´´W	C	CC	Picual	347	6	0.99	0.13	2.99
S40	38 ° 26 ´ 13.654 ´´N	2 ° 44 ´ 8.952 ´´W	O	OC	Picual	4739	9	1.21	0.23	2.76
S41	38 ° 26 ´ 15.772 ´´N	2 ° 43 ´ 58.343 ´´W	O	OT	Picual	4138	8	1.66	0.28	2.78
S42	37 ° 20 ´ 27.258 ´´N	5 ° 15 ´ 53.537 ´´W	C	CT	Picual	3701	6	1.01	0.38	2.89
S43	37 ° 20 ´ 26.495 ´´N	5 ° 15 ´ 54.408 ´´W	C	CC	Picual	1277	6	0.27	0.23	3.02
S44	37 ° 20 ´ 49.089 ´´N	5 ° 16 ´ 5.531 ´´W	O	OC	Picual	4016	6	0.57	0.06	3.00
S45	37 ° 38 ´ 24.949 ´´N	4 ° 29 ´ 7.307 ´´W	O	OT	Picual	2049	10	0.54	0.13	2.96
S46	37 ° 20 ´ 45.233 ´´N	5 ° 16 ´ 37.354 ´´W	C	CT	Picual	2806	7	0.96	0.12	2.92
S47	37 ° 20 ´ 45.998 ´´N	5 ° 16 ´ 38.027 ´´W	O	OT	Gordal	7728	10	1.02	0.22	2.92
S48	37 ° 21 ´ 58.160 ´´N	5 ° 23 ´ 45.887 ´´W	C	CT	Verdial	521	11	1.61	0.24	2.91
S49	37 ° 21 ´ 58.160 ´´N	5 ° 23 ´ 45.887 ´´W	C	CT	Verdial	3063	7	0.36	0.37	2.99
S50	37 ° 16 ´ 17.631 ´´N	5 ° 21 ´ 58.910 ´´W	O	OT	Picual	3336	10	0.62	0.07	2.89
S51	37 ° 18 ´ 2.646 ´´N	5 ° 22 ´ 11.866 ´´W	O	OT	Verdial	3237	6	0.21	0.15	2.99
S52	37 ° 17 ´ 49.307 ´´N	5 ° 23 ´ 6.999 ´´W	C	CT	Gordal	3221	9	1.03	0.05	2.90
S53	37 ° 18 ´ 20.070 ´´N	5 ° 23 ´ 32.298 ´´W	C	CT	Gordal	4400	8	0.55	0.24	2.92
S54	37 ° 25 ´ 18.281 ´´N	5 ° 50 ´ 58.411 ´´W	C	CT	Lechin	7924	10	0.84	0.13	2.98
S55	37 ° 37 ´ 35.704 ´´N	4 ° 4 ´ 15.422 ´´W	C	CT	Verdial	4680	8	1.11	0.19	2.87
S56	37 ° 38 ´ 25.782 ´´N	4 ° 29 ´ 5.570 ´´W	C	CT	Manzanillo	1098	8	0.91	0.26	2.90
S57	37 ° 36 ´ 15.413 ´´N	4 ° 7 ´ 8.734 ´´W	C	CT	Lechin	2071	12	0.58	0.21	3.03
S58	38 ° 8 ´ 0.486 ´´N	3 ° 53 ´ 19.563 ´´W	C	CT	Verdial	1872	8	1.45	0.13	2.94
S59	38 ° 7 ´ 16.381 ´´N	4 ° 51 ´ 34.725 ´´W	C	CC	Picual	2184	8	0.65	0.30	2.96
S60	37 ° 45 ´ 9.285 ´´N	3 ° 37 ´ 7.773 ´´W	C	CC	Picual	274	5	1.00	0.18	2.95
S61	38 ° 10 ´ 35.969 ´´N	4 ° 55 ´ 37.244 ´´W	O	OC	Nevadillo	230	10	2.00	0.23	2.80

	Geographic location									
Orchard	Latitude	Longitude	OMS[a]	SMS[b]	Variety	Abundance	Richness	Shannon	Evenness	PPI
S62	38 ° 10 ´ 35.969 ´´N	4 ° 55 ´ 37.244 ´´W	C	CC	Nevadillo	171	6	1.47	0.46	3.11
S63	38 ° 9 ´ 58.501 ´´N	4 ° 54 ´ 13.709 ´´W	O	OC	Nevadillo	120	7	1.52	0.34	3.28
S64	38 ° 9 ´ 58.501 ´´N	4 ° 54 ´ 13.709 ´´W	O	OC	Nevadillo	209	13	2.08	0.35	2.74
S65	38 ° 9 ´ 25.557 ´´N	4 ° 51 ´ 41.046 ´´W	C	CT	Nevadillo	229	13	2.31	0.48	2.96
S66	38 ° 9 ´ 23.563 ´´N	4 ° 48 ´ 47.867 ´´W	O	OT	Nevadillo	224	8	1.90	0.53	2.68
S67	37 ° 38 ´ 19.563 ´´N	4 ° 29 ´ 7.363 ´´W	C	CT	Nevadillo	560	9	1.16	0.44	2.74
S68	38 ° 9 ´ 23.563 ´´N	4 ° 48 ´ 47.867 ´´W	O	OC	Nevadillo	505	11	1.47	0.27	2.82
S69	38 ° 5 ´ 1.861 ´´N	4 ° 41 ´ 44.308 ´´W	C	CC	Nevadillo	154	6	1.44	0.34	3.01
S70	38 ° 5 ´ 2.202 ´´N	4 ° 41 ´ 45.342 ´´W	O	OC	Nevadillo	974	7	0.61	0.33	2.97
S71	37 ° 45 ´ 9.285 ´´N	3 ° 37 ´ 7.773 ´´W	O	OT	Nevadillo	278	10	2.02	0.14	2.37
S72	37 ° 45 ´ 2.642 ´´N	3 ° 37 ´ 18.547 ´´W	C	CT	Nevadillo	171	8	1.99	0.47	2.71
S73	37 ° 47 ´ 38.368 ´´N	3 ° 26 ´ 44.711 ´´W	O	OC	Picual	4948	11	1.07	0.46	2.95
S74	37 ° 47 ´ 38.368 ´´N	3 ° 26 ´ 44.711 ´´W	O	OT	Picual	695	8	1.28	0.25	2.61
S75	37 ° 47 ´ 37.979 ´´N	3 ° 26 ´ 44.586 ´´W	C	CT	Picual	758	8	0.83	0.29	2.86
S76	37 ° 40 ´ 38.840 ´´N	3 ° 56 ´ 26.233 ´´W	O	OC	Picual	7521	12	1.60	0.19	2.70
S77	37 ° 40 ´ 36.307 ´´N	3 ° 56 ´ 26.528 ´´W	C	CT	Picual	300	7	1.29	0.37	2.99
S78	37 ° 38 ´ 45.700 ´´N	4 ° 30 ´ 9.904 ´´W	O	OC	Picual	89	4	1.31	0.30	2.61
S79	37 ° 33 ´ 38.711 ´´N	4 ° 1 ´ 27.772 ´´W	C	CC	Picual	112	6	1.74	0.30	2.48
S80	37 ° 33 ´ 38.711 ´´N	4 ° 1 ´ 27.772 ´´W	O	OC	Picual	9020	9	0.65	0.40	2.90
S81	37 ° 33 ´ 36.793 ´´N	4 ° 1 ´ 50.570 ´´W	C	CT	Picual	1922	11	0.74	0.15	2.91
S82	37 ° 32 ´ 53.592 ´´N	4 ° 1 ´ 55.925 ´´W	O	OC	Picual	1022	7	1.14	0.17	2.69
S83	37 ° 32 ´ 56.517 ´´N	4 ° 2 ´ 17.564 ´´W	O	OC	Picual	4589	8	0.85	0.26	2.93
S84	37 ° 32 ´ 47.176 ´´N	4 ° 0 ´ 39.959 ´´W	C	CT	Picual	244	6	1.36	0.20	2.77
S85	37 ° 32 ´ 48.041 ´´N	4 ° 0 ´ 37.444 ´´W	O	OC	Picual	885	7	1.52	0.31	2.39
S86	37 ° 33 ´ 20.733 ´´N	4 ° 7 ´ 0.781 ´´W	C	CT	Picual	257	9	1.53	0.35	2.68
S87	37 ° 33 ´ 21.742 ´´N	4 ° 7 ´ 0.430 ´´W	O	OC	Picual	11371	9	0.11	0.35	2.99
S88	37 ° 18 ´ 3.106 ´´N	3 ° 18 ´ 15.740 ´´W	C	CT	Picual	611	7	0.82	0.02	2.90
S89	37 ° 38 ´ 44.508 ´´N	4 ° 30 ´ 11.757 ´´W	O	OC	Picual	682	9	1.42	0.19	2.63
S90	37 ° 17 ´ 41.759 ´´N	3 ° 18 ´ 51.233 ´´W	C	CT	Picual	434	7	1.22	0.33	2.90
S91	37 ° 50 ´ 31.191 ´´N	5 ° 6 ´ 16.573 ´´W	C	CC	Arbequina	88	5	1.29	0.28	2.38

Orchard	Geographic location		OMS[a]	SMS[b]	Variety	Abundance	Richness	Shannon	Evenness	PPI
	Latitude	Longitude								
S92	37 ° 47 ´ 22.197 ´´N	5 ° 0 ´ 30.567 ´´W	C	CC	Arbequina	979	9	0.77	0.27	2.88
S93	37 ° 48 ´ 20.067 ´´N	4 ° 57 ´ 37.666 ´´W	C	CC	Arbequina	1301	5	0.34	0.21	2.98

[a] OMS (Orchard management systems) included: A= Wild olives or 'Acebuches', O= Organic management ,C= Conventional management.

[b] SMS (Soil management systems) included: CC= Conventional management with cover crop, CT= Conventional management with tillage, OC= Organic management with cover crop, and OT= Organic management with tillage.

[c] Number of individuals per 500 cm^3 of soil.
