# Supplementary figures and images for: Soil Properties and Olive Cultivar Determine the Structure and Diversity of Plant-Parasitic Nematode Communities Infesting Olive Orchards Soils in Southern Spain
Source: PLoS One. 2015 Jan 27;10(1):e0116890. doi: 10.1371/journal.pone.0116890 (PMC4308072; doi:10.1371/journal.pone.0116890)

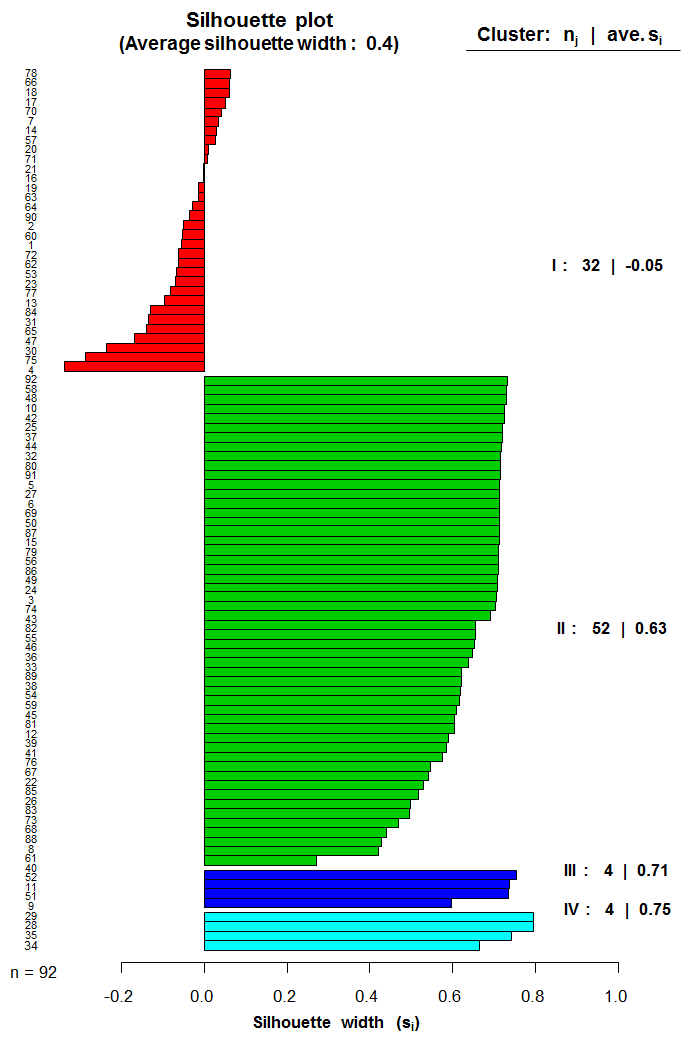

Supplement: S1 Fig — The number of clusters was estimated as that giving the largest average silhouette width for the 92 orchards plots. (TIF) [file pone.0116890.s002.tif]
